# Combination of roflumilast with a beta-2 adrenergic receptor agonist inhibits proinflammatory and profibrotic mediator release from human lung fibroblasts

**DOI:** 10.1186/1465-9921-13-28

**Published:** 2012-03-27

**Authors:** Stacey L Tannheimer, Clifford D Wright, Michael Salmon

**Affiliations:** 1 Respiratory Research, Gilead Sciences, 199 East Blaine St, Seattle, WA 98102, USA

**Keywords:** Roflumilast, PDE4, Beta-2 agonist, Indacaterol, Lung fibroblasts, Anti-inflammatory, Fibrosis

## Abstract

**Background:**

Small airway narrowing is an important pathology which impacts lung function in chronic obstructive pulmonary disease (COPD). The accumulation of fibroblasts and myofibroblasts contribute to inflammation, remodeling and fibrosis by production and release of mediators such as cytokines, profibrotic factors and extracellular matrix proteins. This study investigated the effects of the phosphodiesterase 4 inhibitor roflumilast, combined with the long acting β_2 _adrenergic agonist indacaterol, both approved therapeutics for COPD, on fibroblast functions that contribute to inflammation and airway fibrosis.

**Methods:**

The effects of roflumilast and indacaterol treatment were characterized on transforming growth factor β1 (TGFβ1)-treated normal human lung fibroblasts (NHLF). NHLF were evaluated for expression of the profibrotic mediators endothelin-1 (ET-1) and connective tissue growth factor (CTGF), expression of the myofibroblast marker alpha smooth muscle actin, and fibronectin (FN) secretion. Tumor necrosis factor-α (TNF-α) was used to induce secretion of chemokine C-X-C motif ligand 10 (CXCL10), chemokine C-C motif ligand 5 (CCL5) and granulocyte macrophage colony-stimulating factor (GM-CSF) from NHLF and drug inhibition was assessed.

**Results:**

Evaluation of roflumilast (1-10 μM) showed no significant inhibition alone on TGFβ1-induced ET-1 and CTGF mRNA transcripts, ET-1 and FN protein production, alpha smooth muscle expression, or TNF-α-induced secretion of CXCL10, CCL5 and GM-CSF. A concentration-dependent inhibition of ET-1 and CTGF was shown with indacaterol treatment, and a submaximal concentration was chosen for combination studies. When indacaterol (0.1 nM) was added to roflumilast, significant inhibition was seen on all inflammatory and fibrotic mediators evaluated, which was superior to the inhibition seen with either drug alone. Roflumilast plus indacaterol combination treatment resulted in significantly elevated phosphorylation of the transcription factor cAMP response element-binding protein (CREB), an effect that was protein kinase A-dependent. Inhibition of protein kinase A was also found to reverse the inhibition of indacaterol and roflumilast on CTGF.

**Conclusions:**

These results demonstrate that addition of roflumilast to a LABA inhibits primary fibroblast/myofibroblast function and therapeutically this may impact lung fibroblast proinflammatory and profibrotic mediator release which contributes to small airway remodeling and airway obstruction in COPD.

## Introduction

Fixed airway obstruction in chronic obstructive pulmonary disease (COPD) is characterized by small airway narrowing which can sometimes lead to airway occlusion. This has a profound influence on lung function by reducing the rate of emptying of air from the lungs [1]. Recently it has also been reported that narrowing and loss of small airways proceeds emphysematous destruction in COPD patients [2]. The pathology of small airway disease includes thickening of the airway smooth muscle, increased inflammatory cell recruitment, mucous production and accumulation of fibroblasts/myofibroblasts [3-5]. These resident cells promote inflammation, remodeling and fibrosis by release of inflammatory molecules such as cytokines, production of profibrotic factors, and secretion and deposition of extracellular matrix proteins (ECM).

The novel anti-inflammatory phosphodiesterase-4 (PDE4) inhibitor roflumilast (Daxas^®^; Daliresp™) has recently been approved in the US and EU for GOLD stage 3 and 4 COPD patients. In Europe, Daxas^® ^is indicated for maintenance treatment in severe COPD patients with chronic bronchitis and a history of exacerbations as an add-on to bronchodilator treatment (http://www.fda.gov/downloads/AdvisoryCommittees/CommitteesMeetingMaterials/Drugs/Pulmonary-AllergyDrugsAdvisoryCommittee/UCM207377.pdf). Long acting β2 adrenergic agonists (LABA) have been used as the standard of care for asthma and COPD to provide bronchodilation and symptom relief [6,7]. PDE4 inhibitors and LABA are both known modulators of intracellular cAMP levels in a variety of cells. PDE4 inhibitors maintain baseline levels of cAMP by inhibiting the hydrolysis of cAMP to AMP, while LABA induce high levels of cAMP through a G protein-coupled receptor mechanism [8,9]. Increased cAMP leads to activation of the serine-threonine kinase protein kinase A (PKA), with subsequent activation of the transcription factor cAMP response element-binding protein (CREB) by phosphorylation on Ser-133. This complementary mechanism of action for both compounds suggests that increased or sustained levels of cAMP can lead to alterations in cell functions through CREB-dependent mechanisms, either directly (by binding to cAMP responsive elements (CRE) in the promoter region altering transcription) or indirectly (by association with and sequestration of the cofactor CREB binding protein (CBP)) [10,11].

PDE4 inhibitors have been shown to decrease early stage inflammation and fibrosis in a bleomycin-induced fibrosis model using preventative and therapeutic dosing regimens [12,13]. *In vitro *evidence using roflumilast N-oxide or roflumilast have shown little to modest effects on lung fibroblast profibrotic mediator production and alpha smooth muscle actin (αSMA) expression, while inhibition was significantly augmented indirectly by endogenous PGE_2 _generation [14,15].

Transforming growth factor β1 (TGFβ1) is a well characterized profibrotic molecule that functions in normal wound healing, and can induce differentiation of fibroblasts to myofibroblasts [16]. Myofibroblasts have a more contractile phenotype, express αSMA, secrete ECM proteins and cytokines, and profibrotic molecules like endothelin-1 (ET-1) and connective tissue growth factor (CTGF) [17,18]. Under pathological conditions, excessive production of these factors can lead to lung remodeling and inflammation which may impact lung function [19,20]. In the present studies we employed a TGFβ1-driven model of myofibroblast differentiation to investigate the effects roflumilast in combination with indacaterol on myofibroblast expression and production of ET-1, CTGF, αSMA, and FN as well as release of cytokines in response to proinflammatory cytokine stimulation.

## Materials and methods

### Materials

Reagents used were obtained from: RPMI and BSA (Invitrogen, Carlsbad, CA), TGFβ1 and TNFα (R&D Systems, Minneapolis, MN), H89 and DMSO (Sigma-Aldrich, St. Louis, MO). Compounds were synthesized in Gilead Sciences laboratories.

### Normal human lung fibroblast cell culture

Normal human lung fibroblasts (Lonza Walkersville Inc., Walkersville, MD) were routinely cultured in FGM-2 complete media (Lonza), 5% CO_2_, 37°C, and used at passage 2-5 for experimentation. For experimentation, NHLF were cultured to subconfluence, starved in RPMI + 0.1% BSA overnight, pretreated with compound for 1 h, and stimulated with 10 ng/mL transforming growth factor-β1 (TGFβ1) for 6 h (RNA), 24 h (ET-1 production) or 48 h (FN production and αSMA expression). For experiments with H89, inhibitor was added 30 min before compound pretreatment. All experimental groups were performed in triplicate.

### Transcript quantitation

Cell lysates were generated at 55°C for 30 min (Quantigene Plex 2.0 Custom Reagent System, Affymetrix, Fremont, CA), and run on a Luminex bead-based custom multiplex according to manufacturer's instructions. Median fluorescence intensity (MFI) were generated for each target and normalized to housekeeping genes, peptidylprolyl isomerase B (PPIB) or hypoxanthine phosphoribosyltransferase (HPRT1), which were chosen to match the target transcript abundance. Fold induction was calculated as compared to non-stimulated control.

### Endothelin-1 quantitation

ET-1 production was quantitated by use of QuantiGlo ELISA kit (R&D Systems, Inc.), according to manufacturer's instructions. Results were calculated in pg/mL based on a standard curve, and normalized to cell number.

### Fibronectin quantitation

FN production was quantitated by use of QuantiMatrix ELISA (Millipore), run according to manufacturer's instructions. Results were calculated in ng/mL based on a standard curve, and percentage of control calculated from TGFβ1 stimulated DMSO treated cells.

### α-Smooth muscle actin expression

Cells were trypsinized, fixed (fix buffer 1, 10 min, 37°C, BD Biosciences, San Jose, CA), permeabilized (perm buffer III, 30 min, 4°C, BD Biosciences), stained with an anti-αSMA-FITC antibody (Abcam, Cambridge, MA) and visualized on a LSR II flow cytometer (BD Biosciences). MFI was determined and percentage of control calculated from TGFβ1 stimulated DMSO treated cells.

### pCREB quantitation

Cells were serum starved and were pretreated for 30 min with H89 (10 uM) or DMSO (0.1%), followed by roflumilast and/or indacaterol treatment or DMSO (0.1%) for 30 min, 37°C, washed with cold PBS and then lysed on ice for 20 min (Millipore MAP Cell Signaling kit, Millipore). Lysates were analyzed for pCREB with Luminex bead-based assay (Milliplex MAP Phospho CREB (Ser133) MAPmate, Millipore), as measured by MFI. Results were calculated as percentage of DMSO control.

### CXCL10, CCL5 and GM-CSF secretion

Cells were serum starved, pretreated with compound or DMSO (0.1%, 1 h), stimulated with TNFα (24 h, 10 ng/mL) and cell supernatants run in a Luminex bead-based assay, according to manufacturer's instructions (Millipore). Results were calculated in pg/mL based on a standard curve, and percentage inhibition calculated relative to TNFα-stimulated DMSO control.

### Statistics

IC_50 _data for the effect of indacaterol alone on ET-1 and CTGF protein and transcript levels represents the arithmetic mean ± SEM. IC_50 _data for cytokine inhibition represents the geometric mean with 95% confidence intervals Statistical analysis used a paired two-tailed Student's t-test or repeated one way ANOVA with Tukey's post hoc test. Results where the p-value was < 0.05 were considered significant (GraphPad Software, San Diego, CA).

## Results

### Addition of indacaterol to roflumilast inhibits TGFβ1-induced ET-1

A panel of candidate genes involved in fibrosis and wound healing was assessed in a time- and concentration-dependent manner after TGFβ1 treatment of NHLF. These studies showed that gene induction occurred at 2, 6 and 24 hours after TGFβ1 treatment, but that a 6 hour treatment with TGFβ1 was optimal for maximal gene induction (data not shown). Further characterization of TGFβ1-induced transcripts showed a robust upregulation for CTGF, ET-1 and VEGF (19.4 ± 5.9, 13.6 ± 2.8, 7.5 ± 1.1 fold induced, respectively). Induction of other genes examined was in the 1-3 fold range, with no induction of TNFα or collagen 1A2 (COL1A2) seen (Table 1). Of the three most highly induced genes, VEGF was determined to be insensitive to indacaterol inhibition (data not shown), therefore further characterization of indacaterol and roflumilast inhibition of ET-1 and CTGF was performed.

**Table 1:** TGFβ1-induced gene induction in NHLF (mean fold increase over non-stimulated)

CTGF	TNFAα	ET-1	VEGF	αSMA	TIMP1	TIM3	FN	COL1A1	COL1A2
19.4 ± 5.9	NI	13.6 ± 2.8	7.5 ± 1.1	2.9 ± 0.8	1.5 ± 0.05	3.3 ± 0.7	1.3 ± 0.3	1.9 ± 0.4	NI

RNA was made 6 h after TGFβ1 (10 ng/mL) treatment, and data represents the mean ± SEM of MFI normalized to housekeepers (PPIB, HPRT1), and fold induction calculated versus non-stimulated control (n = 2-3). NI = not induced. Connective tissue growth factor (CTGF), tumor necrosis factor alpha (TNFα), endothelin-1 (ET-1), vascular endothelial growth factor (VEGF), alpha smooth muscle action (αSMA), tissue inhibitors of metalloproteinase (TIMP), fibronectin (FN), collagen 1α1, 1α2 (COL1A1, 1A2).

**Figure 1:**
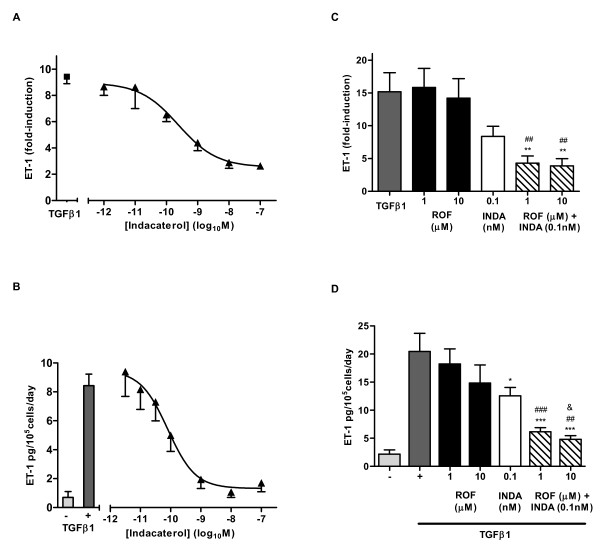
**Effect of roflumilast and indacaterol on TGFβ1-induced ET-1 transcript (A, C) and protein (B, D) from NHLF**. (A, C) NHLF were pretreated with compound or vehicle control for 1 h and RNA was made 6 h after TGFβ1 (10 ng/mL) treatment. Data represents the MFI normalized to HPRT1 and fold induction calculated versus non-stimulated control. All data represent mean ± SEM for n = 3 (A), and n = 8 (C) experiments, run in triplicate. (B, D) NHLF were pretreated with compound or vehicle control for 1 h and conditioned medium was collected 24 h after TGFβ1 (10 ng/mL) treatment and quantitated for ET-1 protein secretion. All data represent mean ± SEM for n = 3 (B), and n = 6 (D) experiments, run in triplicate. ROF: roflumilast, INDA: indacaterol. *p < 0.05, **p < 0.01, and ***p < 0.001 as compared to TGFβ1 vehicle-treated cells, ##p < 0.01 ###p < 0.001 as compared to roflumilast treated cells, &p < 0.05 as compared to indacaterol treated cells.

TGFβ1 stimulation of NHLF resulted in an upregulation of ET-1 transcript levels of 9.4 ± 0.5 fold compared to non-stimulated control cells. Evaluation of the LABA indacaterol demonstrated a concentration-dependent inhibition of ET-1, with an IC_50 _of 0.2 nM and maximal inhibition of 72.0 ± 0.3% (Figure 1A). ET-1 protein production was induced by TGFβ1 treatment, with a change from 0.7 ± 0.4 pg/mL to 8.4 ± 0.8 pg/mL for non-stimulated versus TGFβ1 stimulated NHLF, respectively. Indacaterol inhibited protein production in a concentration-dependent manner with an IC_50 _of 80.6 pM, and concentrations of 10 nM indacaterol and above driving levels of ET-1 down to baseline (Figure 1B). Based on these findings for indacaterol on both ET-1 transcript and protein levels, a concentration of 0.1 nM indacaterol was chosen that gave an IC_30-40 _to further characterize combination treatment with roflumilast.

For combination treatment studies, TGFβ1 stimulation increased ET-1 transcript levels by 15.2 ± 2.9 fold compared to non-stimulated controls. Neither roflumilast nor indacaterol (0.1 nM) alone led to a statistically significant inhibition of ET-1 transcript, although there was a trend for inhibition with indacaterol. When assessed together, indacaterol (0.1 nM) plus 1 or 10 uM roflumilast combination treatment demonstrated 71.7 ± 1.1% (p < 0.01) and 74.5 ± 1.1% (p < 0.01) inhibition respectively, which was greater than either agent alone (Figure 1C).

Further characterization of ET-1 protein production demonstrated an increase from 2.2 ± 0.7 pg/mL to 20.5 ± 3.2 pg/mL for non-stimulated versus TGFβ1 stimulated NHLF. Roflumilast treatment alone (1 and 10 μM) inhibited ET-1 secretion by 11.1 ± 2.7% and 27.6 ± 3.2% of control, respectively, while indacaterol (0.1 nM) inhibited by 43.3 ± 1.5% (p < 0.05). Similar to the results observed with ET-1 transcript levels, when indacaterol (0.1 nM) was added to roflumilast treatment (1 and 10 μM), the inhibition of ET-1 protein secretion was more pronounced than with either agent alone, with inhibition of 78.3 ± 4.0% (p < 0.001) and 85.7 ± 3.6% (p < 0.001) of control, respectively (Figure 1D).

### Addition of indacaterol to roflumilast inhibits TGFβ1-induced CTGF in a PKA-dependent manner

**Figure 2:**
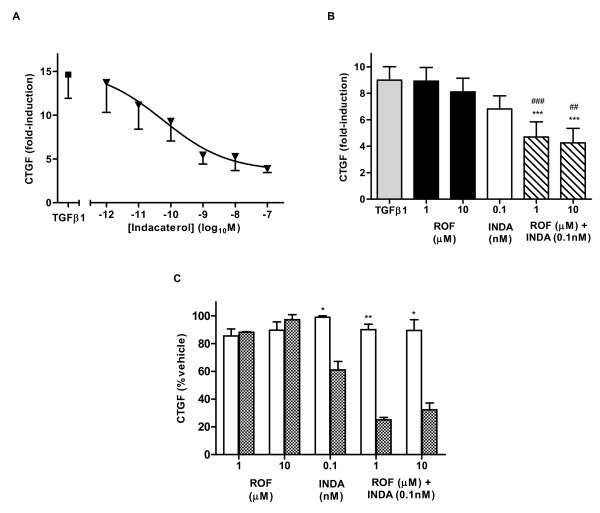
**Roflumilast and indacaterol inhibition of CTGF transcript is PKA-dependent**. For all experiments, NHLF were pretreated with compound or vehicle control for 1 h and RNA was made 6 h after TGFβ1 (10 ng/mL) treatment. (C) For experiments with H89 (10 μM), inhibitor was added 30 min before compound pretreatment, with open bars (+H89) and checked bars (-H89) treatment. All data represents the MFI normalized to PPIB and fold induction calculated versus non-stimulated control. The data represent the mean ± SEM for n = 5 (A), n = 8 (B) and n = 3 (C) experiments, run in triplicate. ROF: roflumilast, INDA: indacaterol. (B) ***p < 0.001 as compared to TGFβ1 vehicle-treated cells, ##p < 0.01 and ###p < 0.001 as compared to roflumilast treated cells. (C) *p < 0.05, **p < 0.01 as compared to H89 negative control.

TGFβ1 stimulation caused an upregulation of CTGF transcript levels of 14.6 ± 2.7 fold compared to non-stimulated controls. Indacaterol demonstrated a concentration-dependent inhibition of CTGF, with an IC_50 _of 57.4 pM and maximal inhibition of 73.5 ± 0.4%. Indacaterol at 0.1 nM caused 36.6 ± 2.2% inhibition, within the same range seen for ET-1 transcript and protein inhibition at this concentration (Figure 2A).

In combination studies, CTGF transcript levels were upregulated 9.0 ± 1.0 fold following TGFβ1 stimulation. Roflumilast alone caused a 9.9 ± 1.0% decrease in CTGF transcript at the highest concentration tested (10 μM). Indacaterol (0.1 nM) had no significant effect alone, but in combination with roflumilast at both 1 or 10 μM caused a significant decrease in transcript levels compared to control, with 47.8 ± 1.1% (p < 0.001) and 52.7 ± 1.1% (p < 0.001) inhibition, respectively (Figure 2B).

To interrogate the mechanism of action for the superior inhibition seen with roflumilast plus indacaterol treatment, addition of the PKA inhibitor H89 was evaluated. As previously seen, roflumilast alone produced very little inhibition of CTGF transcript levels and the addition of H89 had not effect (Figure 2C). Indacaterol (0.1 nM) led to inhibition of CTGF by 38% and this was completely reversed by H89 treatment (p < 0.05). Indacaterol (0.1 nM) in combination with roflumilast strongly inhibited CTGF, an effect that was almost completely blocked by H89 addition, with 90.1 ± 3.8% (p < 0.01) and 89.4 ± 7.9% (p < 0.05) of control at 1 and 10 μM, respectively.

### Indacaterol and roflumilast plus indacaterol increase phosphorylated CREB

**Figure 3:**
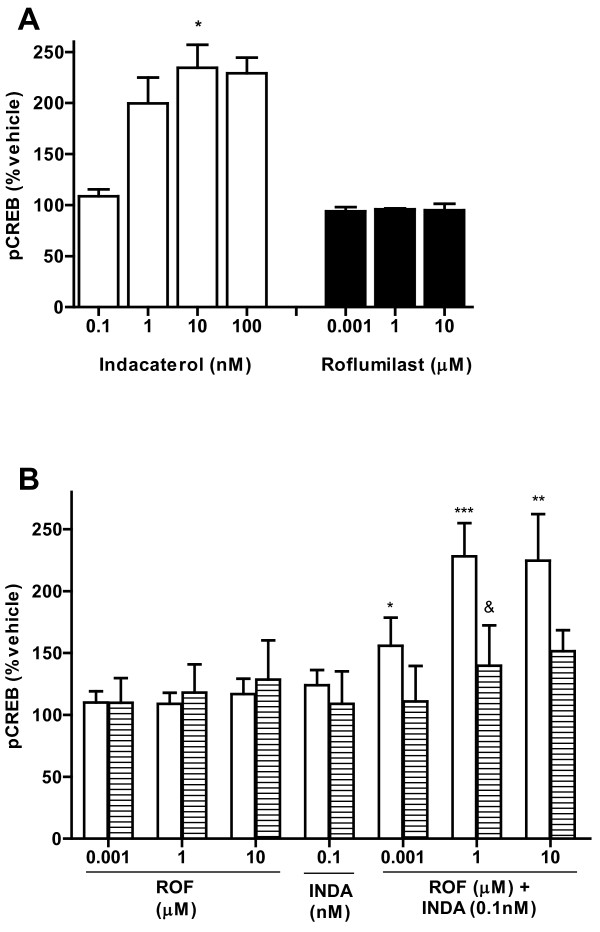
**Indacaterol and roflumilast increased pCREB in a PKA-dependent manner**. NHLF were treated with compound (30 min) (A), or pretreated with - (open bars) or + H89 (hatched bars) followed by compound treatment (30 min) (B), and MFI for pCREB determined. Data is reported as percentage of vehicle control, mean ± SEM from n = 4-6 experiments. ROF: roflumilast, INDA: indacaterol. * p < 0.05, **p < 0.01, ***p < 0.001 compared to vehicle treated cells, &p < 0.05 as compared to H89 negative control.

Roflumilast treatment (0.001-10 μM) resulted in no increased pCREB at any concentration tested (Figure 3A). Indacaterol treatment however caused a concentration-dependent increase in pCREB, with 10 nM indacaterol eliciting an increase of 134.6 ± 22.4% above control (p < 0.05). Further characterization of roflumilast treatment again showed little effect on pCREB (maximal 16.8% above control), while indacaterol (0.1 nM) increased pCREB with a 23% increase over control (Figure 3B). When 0.1 nM indacaterol was combined with roflumilast at 0.001, 1 or 10 μM, pCREB was increased by 56 ± 23% (p < 0.05), 128 ± 27% (p < 0.001) and 125 ± 38% (p < 0.01) over DMSO control cells, respectively. Use of H89 reduced pCREB to near basal levels as compared to no H89 treatment.

### Modulation of αSMA expression and FN secretion in NHLF by roflumilast and indacaterol combination

**Figure 4:**
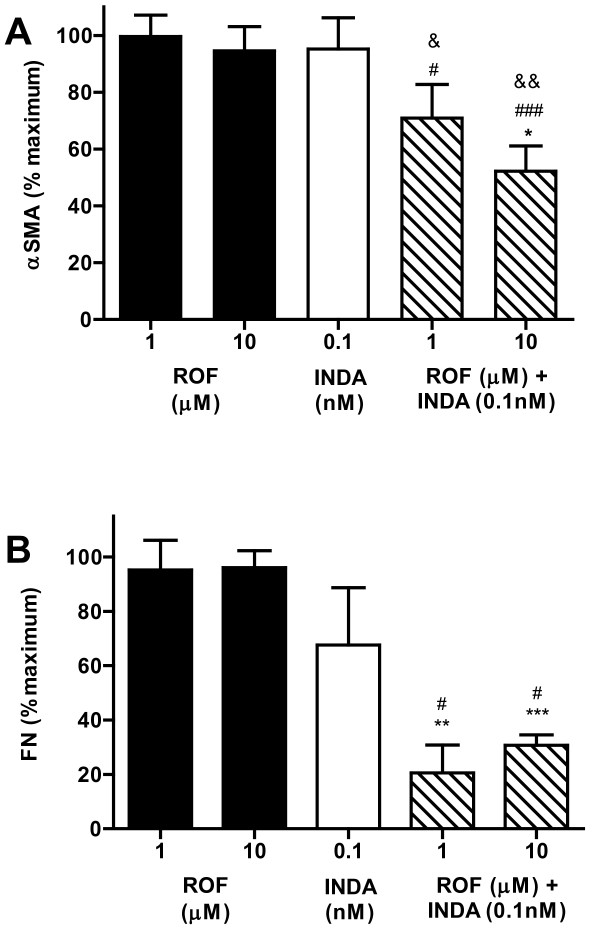
**Effect of roflumilast and indacaterol on TGFβ1-induced αSMA expression and FN secretion in NHLF**. (A) NHLF were pretreated with compound or vehicle control for 1 h, treated with TGFβ1 (10 ng/mL) for 48 h, and αSMA expression quantitated by flow cytometry. Data is reported as the percentage of TGFβ1 vehicle-treated cells, mean ± SEM for n = 4. (B) NHLF were pretreated with compound or vehicle control for 1 h, treated with TGFβ1 (10 ng/mL) for 48 h and FN secretion quantitated. Data is reported as the percentage of TGFβ1 vehicle-treated cells, mean ± SEM for n = 4-6. ROF: roflumilast, INDA: indacaterol. *p < 0.05, **p < 0.01, ***p < 0.001 as compared to TGFβ1 vehicle-treated cells, #p < 0.05 and ###p < 0.001 as compared to roflumilast treated cells, &p < 0.05 and &&p < 0.01 as compared to indacaterol treated cells.

After TGFβ1 treatment, fibroblasts acquire a more contractile phenotype, and this is characterized by expression of αSMA, accompanied by an increased potential to secrete ECM proteins. Treatment of NHLF with TGFβ1 caused a 10.4 ± 5.5 fold upregulation of αSMA expression. Roflumilast (1 and 10 μM) and indacaterol (0.1 nM) alone failed to inhibit αSMA expression (Figure 4A). In combination, roflumilast plus indacaterol inhibited αSMA expression 29.1 ± 11.8% and 47.8 ± 8.9% (p < 0.05) of control at 1 and 10 μM roflumilast plus indacaterol (0.1 nM), respectively.

Evaluation of the production of the ECM protein FN showed that after treatment of NHLF with TGFβ1, FN levels increased from 4580 ± 2029 ng/mL to 8700 ± 3693 ng/mL. Treatment with either roflumilast (1 or 10 μM) or indacaterol (0.1 nM) alone caused no significant inhibition of FN secretion (Figure 4B). Roflumilast (1 or 10 μM) in combination with indacaterol (0.1 nM) however demonstrated inhibition of 79.4 ± 10.2% (p < 0.01) and 69.3 ± 3.8% (p < 0.001) of control respectively.

### Characterization of roflumilast and indacaterol inhibition on TNFα-induced CXCL10, CCL5 and GM-CSF secretion

**Figure 5:**
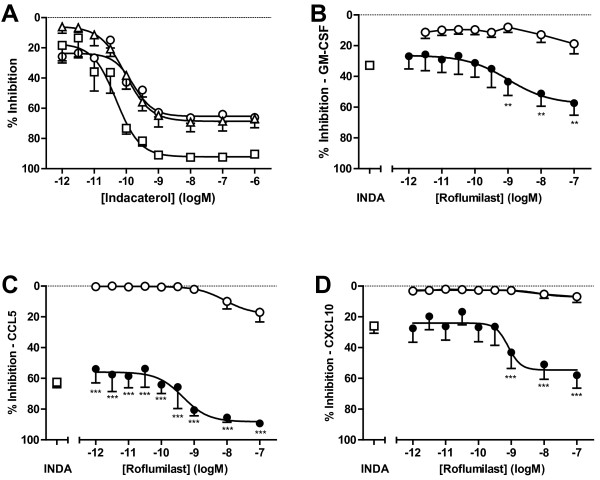
**Indacaterol, roflumilast and combination effects on TNFα -induced cytokines GM-CSF, CCL5 and CXCL10 from NHLF**. (A) Data represents the mean ± SEM inhibition of indacaterol for GM-CSF (open circle), CCL5 (open square) or CXCL10 (open triangle) released from NHLF compared to vehicle treated cells following stimulation with TNFα (10 ng/mL, 24 h), n = 6. (B-D) Data represents the mean ± SEM inhibition of GM-CSF (B), CCL5 (C) or CXCL10 (D) released from NHLF compared to vehicle-treated cells following stimulation with TNFα (10 ng/mL, 24 h). The open circles represent roflumilast (n = 8 donors), open square represents indacaterol at 0.1 nM (n = 8 donors) and closed circles represent roflumilast in the presence of 0.1 nM indacaterol (n = 4 donors). INDA: indacaterol. **p < 0.01, ***p < 0.001 as compared to roflumilast alone.

Conditioned media from TNFα- treated NHLF was assessed on a panel of cytokines, where GM-CSF, CCL5 and CXCL10 were found to be increased to 228 ± 12, 21042 ± 513, and 6220 ± 241 pg/mL at 24 h post-stimulation, respectively. Roflumilast or indacaterol alone, and in combination, were assessed for their ability to inhibit TNFα-induced production of these three cytokines from NHLF. Indacaterol caused a potent concentration-dependent inhibition of all three cytokines, with IC_50 _values of 0.1 nM (CI 0.03-0.4 nM), 0.02 nM (CI 0.007-0.06 nM), and 0.09 nM (CI 0.04-0.2 nM) for GM-CSF, CCL5 and CXCL10, respectively (Figure 5A). A submaximal concentration of 0.1 nM indacaterol was chosen to assess the effects of roflumilast in combination. Roflumilast alone showed little to modest effects on the inhibition of GM-CSF, CCL5 and CXCL10, with no more than 20% inhibition seen up to 0.1 μM (Figure 5B-D). Roflumilast in combination with 0.1 nM indacaterol caused a concentration-dependent inhibition of all three cytokines, greater than roflumilast treatment alone. In combination the maximal inhibition for GM-CSF shifted from 18.8 ± 6.6% to 57.5 ± 7.8% (p < 0.01), for CCL5 from 17.1 ± 6.2% to 89.4 ± 2.5% (p < 0.001), and for CXCL10 from 7.0 ± 3.8% to 58.1 ± 8.4% (p < 0.001), as compared to roflumilast alone. The IC_50 _for roflumilast plus indacaterol (0.1 nM) treatment was 1.0 nM (CI 0.1 - 7.0 nM), 0.2 nM (CI 0.1 - 0.3 nM) and 0.4 nM (CI 0.07 - 2.2 nM) for GM-CSF, CCL5 and CXCL10, respectively.

## Discussion

The use of the PDE4 inhibitor roflumilast provides a novel therapeutic option that improves lung function in COPD patients, and preclinically has been shown to produce a variety of anti-inflammatory effects [21-23]. While some studies have begun to address the effects that roflumilast has on resident lung cells, our studies extend those findings by examining the effect roflumilast may have when added onto existing β_2 _agonist treatment in the context of small airway disease.

TGFβ1 has been well characterized on fibroblasts for its effects in wound healing [24], but excessive levels of TGFβ1, as seen in chronic disease states, is likely to contribute to the inflammation and remodeling seen in small airway disease. Indeed, increased production of TGFβ1 from the epithelium in the small airways of COPD patients has been seen and correlates with small airway obstruction [25]. *In vivo*, the intratracheal administration of TGFβ1 led to increased collagen deposition in the airways and airway hyperreactivity [26]. TGFβ1-driven fibrosis can be further amplified by the direct induction of other profibrotic molecules, ET-1 and CTGF [17,18,27,28]. Antagonists of ET-1 have been evaluated in clinical trials for use in idiopathic pulmonary fibrosis (IPF) with limited success, but there is clearly a biological rationale for ET-1 in pulmonary fibrosis which still supports antagonism and/or decreased levels of protein as providing a potential therapeutic advantage [29,30]. Our studies show that indacaterol caused a concentration-dependent inhibition of ET-1 and CTGF. When a submaximal concentration of indacaterol was combined with roflumilast there was an augmentation of inhibition of ET-1 transcript levels and protein production, with a similar pattern of inhibition on CTGF transcript levels. The fact that roflumilast plus indacaterol combination treatment reduced TGFβ1-driven increases in ET-1 and CTGF provides strong evidence for effects on profibrotic mediators upstream of therapeutic interventions with ET-1 and/or CTGF antagonists. This combination treatment could lead to more pronounced effects on profibrotic mediator release because of the broad effect it appears to have on key mediators involved in this process.

Previous studies looking at the effects of roflumilast on anti-remodeling events have demonstrated a modest effect on collagen gel contraction and FN secretion from collagen embedded NHLF [15]. While in our studies high concentrations of roflumilast had little effect, our results are in agreement with the observation that when roflumilast is combined with other agents that can elevate cAMP, an enhanced inhibition of mediators involved in fibroblast function is observed [14]. While PGE_2 _can lead to enhanced inhibition of PDE4 inhibitors on fibroblast function, due to either increased endogenous PGE_2 _production or exogenous addition [14,15], in our assay system using NHLF we saw no direct increase in PGE_2 _to point to this as a possible mechanism of action for enhanced inhibition (data not shown).

TGFβ1 can signal downstream of its receptor through activation of the transcription factor family of Smad proteins [31]. Evaluation of cAMP elevating agents that can inhibit TGFβ-induced pathways have shown that Smad phosphorylation and translocation to the nucleus remain intact, with the mechanism of action being independent of direct effects on Smad signaling [10,32]. In the present study, we showed that roflumilast plus indacaterol combination treatment elevates pCREB in NHLF and the phosphorylation is dependent on activation of PKA, as demonstrated by use of the PKA inhibitor H89. We go on to extend these findings and show that the inhibitory affect of indacaterol and roflumilast plus indacaterol combination treatment on CTGF transcript levels is PKA-dependent as well. While some effects of pCREB are due to CRE binding, others have shown that the association of pCREB with CREB-binding protein (CBP)/p300 serves as a limiting cofactor for other transcription factors such as Smad 3/4 and NF-κB [10,11,33]. While the cAMP/PKA pathway is a pivotal activator of pCREB, there is also evidence for downstream effects on both Raf-1 and ERK1/2 phosphorylation [34]. A more extensive evaluation of the mechanism of action downstream of pCREB would need to be done in order understand the inhibition observed in our studies.

Fibroblasts can both respond to, and secrete proinflammatory cytokines. The well characterized proinflammatory cytokine, TNFα, in our model system induced secretion of CXCL10, CCL5 and GM-CSF. CXCL10 and CCL5 are chemokines that are important for monocyte and T cell recruitment to the lung, and GM-CSF is responsible for myeloid survival, differentiation and activation [35,36]. CCL5 has been found to be upregulated in the airways and sputum of COPD patients during exacerbations [37], while CXCL10 levels have been seen to be elevated in the lungs of COPD patients [38]. The ability to inhibit the secretion of these cytokines from resident lung fibroblasts is likely to impact the accumulation of immune cells in the local environment, and lead to further remodeling of the small airways. CXCL10, CCL5 and GM-CSF were all sensitive to indacaterol inhibition in a concentration-dependent manner. Addition of roflumilast to a submaximal concentration of indacaterol further increased levels of cytokine inhibition, more than either agent alone. There is most likely a difference in the regulatory mechanisms driving the inhibition of these three different cytokines in NHLF, especially since CCL5 inhibition can be driven almost completely to baseline levels, and the CCL5 promoter region contains a NF-κB binding site and a CRE [39]. It has previously been shown that cAMP elevators, PGE_2 _and forskolin, can reduce CCL5 production in a H89-dependent manner [40]. While we did not directly investigate the mechanism of action for inhibition with roflumilast plus indacaterol combination, there is likely to be an overlap of mechanisms that works in tandem to give better than additive effects.

We are the first to report the characterization of the combination of a PDE4 inhibitor with a LABA on primary normal human lung fibroblasts, and demonstrate an enhanced effect by addition of a PDE4 inhibitor to a low concentration of beta-2 agonist on inhibition of NHLF cell function as they relate to proinflammatory and profibrotic mechanisms. It is possible therefore that therapeutically the addition of roflumilast to LABA treatment could impact lung fibroblast proinflammatory and profibrotic functions in the small airways, a process which contributes to fixed airway obstruction in COPD.

## Abbreviations

PDE4: phosphodiesterase 4; COPD: chronic obstructive pulmonary disease; LABA: long-acting β_2 _adrenoceptor agonist; TGFβ1: transforming growth factor-β1; ET-1: endothelin-1; CTGF: connective tissue growth factor; αSMA: alpha smooth muscle actin; FN: fibronectin; TNFα: tumor necrosis factor-α; pCREB: phosphorylated cAMP response element-binding protein; CCL5: chemokine (C-C motif) ligand 5; CXCL10: chemokine (C-X-C motif) ligand 10; GM-CSF: granulocyte macrophage colony-stimulating factor; PKA: protein kinase A.

## Competing interests

The authors declare that they have no competing interests.

## Authors' contributions

ST and MS developed the hypothesis, ST conducted the study design, performance of experiments, analysis and interpretation of the data and manuscript preparation. CW and MS helped in the study design, interpretation of the data and manuscript preparation. All authors have read and approved the final manuscript.
